# Do individual or organizational factors influence cultural competency of maternal newborn nurses?: a cross-sectional study

**DOI:** 10.4069/whn.2024.11.03

**Published:** 2024-12-30

**Authors:** Semi Lee, Hyunkyung Choi

**Affiliations:** 1 Delivery Room, Pohang Women’s Hospital, Pohang, Korea; 2 College of Nursing & Research Institute of Nursing Innovation, Kyungpook National University, Daegu, Korea

**Keywords:** Attitude, Cultural competency, Cultural diversity, Nurses, Pregnant women

## Abstract

**Purpose:**

Cultural competency is a very important ability of nurses in women’s hospitals in providing nursing care during pregnancy and childbirth. This study explored how multicultural attitudes, multicultural efficacy, intercultural communicative competency, and hospital support for cultural competency influence the cultural competency of nurses in women’s hospitals.

**Methods:**

A cross-sectional correlational study design was used. The study involved 150 nurses from five women’s hospitals located in Gyeongsangbuk-do and Gyeongsangnam-do, South Korea. Participants completed a packet of structured self-report questionnaires, which included the Korean version of the Cultural Competence Scale for Clinical Nurses, the Multicultural Attitude Scale Questionnaire, the Intercultural Communicative Competence Questionnaire, the Transcultural Self-Efficacy Scale, and the Organizational Support among Cultural Competence Assessment Instrument. We analyzed the collected data using descriptive statistics, the t-test, one-way analysis of variance, Pearson correlation coefficients, and hierarchical multiple regression analysis.

**Results:**

Among the general characteristics, educational level, religion, and experience with overseas travel were identified as factors influencing cultural competency. In the final model, multicultural attitudes (β=.46, *p*<.001) and intercultural communicative competency (β=.19, *p*=.025) emerged as significant individual factors that affected cultural competency. This model accounted for 49.8% of the variance in cultural competency.

**Conclusion:**

This study identified multicultural attitudes and intercultural communicative competency as significant individual factors contributing to the cultural competency of nurses in women’s hospitals. Therefore, enhancing these nurses’ multicultural attitudes and intercultural communicative competency is essential for improving their overall cultural competency.

## Introduction

In recent years, Korea has experienced an increase in its foreign resident population, driven by higher rates of international marriages, an influx of foreign labor, and the popularity of the Korean Wave (Hallyu); these individuals now make up 4.3% of the total population [1]. This percentage is nearing the OECD (Organization for Economic Co-operation and Development) countries’ 5% threshold for defining a multicultural society, indicating that Korea is rapidly evolving into a more multicultural nation. The 2022 Yearbook of Korea immigration statistics, published by the Ministry of Justice, reports that approximately 166,000 marriage immigrants were living in Korea in 2022 [1]. Furthermore, in that same year, marriages between Korean men and foreign women accounted for 72% of all international marriages. Within 2 years of marriage, 60.8% of these women gave birth to their first child in a hospital or clinic [1]. Given Korea’s low birth rate, which is a significant social concern, the increase in women migrating to Korea for marriage and the births within these multicultural families underscore the urgent need for culturally competent nursing care during pregnancy and childbirth.

Marriage immigrant women (MIW) often encounter cultural and language barriers when they relocate to Korea. These challenges, combined with the physical discomfort and emotional instability associated with pregnancy and childbirth, can increase their psychological vulnerability, particularly as they are also at risk of social isolation [2]. Therefore, it is crucial for nurses who care for pregnant MIW to have cultural competency. This competency is essential because the health of pregnant women is directly linked to fetal health. Research has indicated that pregnant MIW are less likely to attend prenatal check-ups compared to their Korean counterparts [3] and have higher incidences of meconium-stained amniotic fluid and low birth weight infants [4].

Cultural competency involves recognizing one’s own cultural background and respecting differences in culture [5]. In the field of nursing, it specifically refers to the ability to respect and understand the diverse cultural backgrounds of patients, while effectively responding to their unique needs [6]. Enhancing cultural competency among nurses is vital in a multicultural society, offering benefits to both nurses and patients. This is especially critical for MIW, who are often more susceptible to crises due to cultural differences experienced during pregnancy and childbirth, potentially leading to significant health disparities; therefore, it is imperative to develop nurses’ cultural competency to better advocate for these women [7]. A study conducted by Cross [8] indicated that cultural competency is shaped by both the individual characteristics of the nurse and the organizational factors within the healthcare setting. Consequently, it is essential to identify the individual and organizational factors that influence the cultural competency of nurses in women’s hospitals and utilize these insights to enhance cultural competency.

Among individual factors, multicultural attitudes contribute to an accurate understanding of and empathy towards diverse cultures, facilitating consistent strategies in addressing multicultural issues [9]. Nursing that embraces multiculturalism not only improves the satisfaction of multicultural patients but also helps reduce health disparities linked to cultural differences [6]. Therefore, a multicultural attitude is vital for nurses who care for multicultural patients.

Multicultural efficacy refers to the confidence to proactively engage with multicultural environments [10]. It represents another individual factor that may influence cultural competency. Individuals with high multicultural efficacy recognize and respect cultural differences, displaying both confidence and practical competency in multicultural interactions [11]. Conversely, those with low multicultural efficacy might face cultural conflicts or form stereotypes about foreign patients [11]. Consequently, it is essential to evaluate the multicultural efficacy of nurses in women’s hospitals and examine its effect on their cultural competency when providing care to patients from diverse backgrounds.

Intercultural communicative competency is a key individual factor that impacts cultural competency, directly influencing both nurses and patients in multicultural care settings. A prior study has found that many MIW face language barriers when seeking healthcare services in Korea, hindering their ability to receive proper care [12]. Healthcare providers also face challenges in gathering health-related information, such as family and medical histories, and frequently deal with misunderstandings and confusion, which disrupt the continuity of care [13].

Among organizational factors, hospital support for cultural competency is anticipated to impact nurses’ cultural competency. This support encompasses cultural competency training for staff, culturally relevant printed materials and forms, and hospital policies that embrace cultural diversity. Without sufficient hospital support for multicultural patients, nurses may struggle to provide appropriate care due to the added responsibilities of their roles [14]. In turn, this lack of support can heighten the psychological burden on nurses. Thus, hospital support for cultural competency is essential in delivering care to multicultural patients. Although it is expected that such support would positively affect nurses’ cultural competency, research exploring this relationship remains insufficient.

Previous research on cultural competency in Korean nursing has predominantly focused on nurses working in general hospitals [15-18]. Many MIW tend to initially settle in smaller cities and seek care at women’s hospitals during pregnancy and childbirth. However, there has been limited research on the cultural competency of nurses in women’s hospitals located in these provincial cities, where cultural support services are less readily available than in major urban centers. Moreover, there is a scarcity of systematic studies in Korea that differentiate cultural competency into individual and organizational factors. Consequently, this study aimed to examine women’s hospital nurses’ multicultural attitudes, multicultural efficacy, and intercultural communicative competency as individual factors and the hospital’s support for cultural competency as an organizational factor to determine the influence of these variables on cultural competency. The findings of this study are intended to provide foundational data for the development of educational programs aimed at enhancing the cultural competency of nurses in women’s hospitals.

## Methods

**Ethics statement** This study was approved by the Institutional Review Board of Kyungpook National University (No. 2023-0067). Informed consent was obtained from the participants.

This correlational cross-sectional study investigated the effects of multicultural attitudes, multicultural efficacy, intercultural communicative competency, and hospital support for cultural competency on the cultural competency of nurses in women’s hospitals. The study adhered to the STROBE guidelines (https://www.strobe-statement.org/).

### Participants

A convenience sample of 150 nurses was selected from five women’s hospitals in Gyeongsangbuk-do and Gyeongsangnam-do, Korea. These hospitals had bed capacities ranging from 51 to 135. The participants were nurses who worked in delivery rooms, neonatal units, or obstetric wards and had experience caring for MIW. All participants were informed about the study’s purpose and provided written consent. Nurses in administrative roles who did not provide direct patient care were excluded from the study. The required sample size was determined using the G*Power program (ver. 3.1.9.4). Previous research on nurses’ cultural competency [19] suggested that, for a multiple regression analysis, at least 135 participants were needed. This calculation was based on a significance level of 0.05, a medium effect size of 0.15, a power of 0.80, and 14 predictors. To account for a potential 10% dropout rate, 150 nurses were recruited. The predictor variables consisted of four independent variables and 10 general characteristics previously identified in studies [15-17,20-22]. These characteristics included age, marital status, education level, religion, position in the hospital, working unit, overseas travel experience, having a foreign friend, previous multicultural education, and the availability of guidelines for caring for foreign patients in the hospital. Participation in the study was voluntary. Participants were informed that they could withdraw at any time without any disadvantage. All 150 distributed questionnaires were collected and used for the final data analysis.

### Measurements

All tools used in this study were approved for use by their developers, translators, and modifiers.

#### Cultural competency

Cultural competency was measured using the Korean Version of the Cultural Competence Scale for Clinical Nurses, which was developed by Chae and Lee [23]. This instrument includes 33 items divided into four subdomains: cultural awareness, cultural knowledge, cultural sensitivity, and cultural skills. Each item is evaluated on a 7-point Likert scale (1, strongly disagree to 7, strongly agree). The total scores can range from 33 to 231, with higher scores indicating greater cultural competency of understanding and respect for patients’ cultural differences. When initially developed, the tool showed a Cronbach’s α of .93; and in this study, it was .94.

#### Individual factors

##### 1) Multicultural attitudes

To measure multicultural attitudes, this study utilized the Multicultural Attitude Scale Questionnaire, which was adapted for Korean college students by Kang and Lim [9] from Munroe and Pearson’s original tool [24]. The questionnaire comprises 16 items divided into three subdomains: recognizing differences, openness and acceptance, and commitment. Each item is rated on a 6-point Likert scale (1, strongly disagree to 6, strongly agree), with total scores ranging from 16 to 96. Higher scores indicate higher multicultural attitudes, which is more empathy and a stronger commitment to addressing cultural inequities experienced by individuals from diverse cultures. Initially, Cronbach’s α values for this tool were .78 for recognizing differences, .76 for openness and acceptance, and .78 for commitment [9]. In the current study, Cronbach’s α values were .74 for recognizing differences, .75 for openness and acceptance, and .83 for commitment, resulting in an overall Cronbach’s α of .89.

##### 2) Multicultural efficacy

Multicultural efficacy was measured using the Transcultural Self-Efficacy Scale, developed by Oh et al. [25]. This scale is derived from Jeffreys’ Transcultural Self-Efficacy Tool [26] and includes 25 items distributed across three subdomains: cognitive, practical, and affective. Each item is rated on a 4-point Likert scale (1, not at all confident to 4, very confident). The total scores range from 25 to 100, with higher scores reflecting greater multicultural efficacy, characterized by both proactivity and confidence in multicultural environments. The Cronbach’s α at the time of the tool’s development was .88 [26]; and in this study, it was .90.

##### 3) Intercultural communicative competency

To assess intercultural communicative competency, we utilized the Intercultural Communicative Competence Questionnaire, originally developed by Lee [27] and later modified by Lee and Kim [28]. This instrument comprises four subdomains: communication skills, knowledge, attitudes, and awareness. For the purposes of this study, we selected 10 items specifically related to intercultural communication skills to evaluate the communication competency of nurses in clinical settings. These items were scored using a 5-point Likert scale (1, strongly disagree to 5, strongly agree), with total scores ranging from 10 and 50. Higher scores denote a greater level of intercultural communicative competency, indicative of an improved ability to engage effectively with multicultural patients and to navigate communication barriers. The reliability of the questionnaire was confirmed with a Cronbach’s α of .84 at its inception, .96 in the study by Lee and Kim [28], and .84 in the current study.

#### Organizational factor

##### 1) Hospital support for cultural competency

Hospital support for cultural competency was assessed using the organizational support section of the Cultural Competence Assessment Instrument, which was developed by Balcazar et al. [29] and later translated by Choi et al. [30]. This tool includes eight items, each rated on a 4-point Likert scale (1, strongly disagree, to 4, strongly agree), with possible total scores between 8 and 32. For the purposes of analysis, three negatively worded items (items 5, 6, and 8) were reverse-coded. Higher scores reflect greater hospital support for cultural competency, including efforts such as providing multicultural education for nurses, integrating cultural competency into hospital policies and systems, and supplying the necessary resources and forms for multicultural patients. The reliability of this tool, as measured by Cronbach’s α, was .80 at the time of its development [29], .74 in Korea [30], and .71 in the current study.

### Data collection

Data collection for this study took place from July 18 to August 18, 2022. Before initiating the survey, the researchers explained the study’s objectives and methods to the heads of each hospital and obtained permission for data collection. The researcher then visited the departments of the study participants to explain the study’s objectives and procedures. After obtaining written consent, the researcher distributed questionnaires to those who agreed to participate. Upon completion, participants sealed their questionnaires in individual envelopes to ensure confidentiality, and the researcher collected them during a follow-up visit. Additionally, participants received a small gift as a token of appreciation.

### Data analysis

Data were analyzed using IBM SPSS for Windows ver. 25.0 (IBM Corp., Armonk, NY, USA). Descriptive statistics assessed participants’ general characteristics, multicultural attitudes, multicultural efficacy, intercultural communicative competency, hospital support for cultural competency, and levels of cultural competency. We examined differences in cultural competency based on participants’ general characteristics using t-tests and one-way analysis of variance, with *post hoc* analyses conducted using Scheffé test. Pearson correlation coefficients were calculated to explore the relationships among cultural competency, multicultural attitudes, multicultural efficacy, intercultural communicative competency, and hospital support for cultural competency. Hierarchical multiple regression analysis was used to determine the effects of multicultural attitudes, multicultural efficacy, intercultural communicative competency, and hospital support for cultural competency on nurses’ cultural competency.

## Results

### General characteristics of participants

The average age of participants was 37.15±8.42 years, with the majority, 38% (n=57), being in their 30s. Among the participants, 56.7% were married. Educational attainment was distributed as follows: 52.0% had an associate degree, 41.3% held a bachelor’s degree, and 6.7% had earned a graduate degree. Additionally, 61.3% reported no religious affiliation. In terms of employment, 80.0% of the participants were staff nurses. The distribution across different wards was: delivery room (36.0%), neonatal unit (32.7%), and obstetric ward (31.3%). Regarding multicultural experiences, 86.0% had traveled abroad, 10.7% had foreign friends, and 31.3% had undergone multicultural education. Moreover, 82.0% indicated that their hospital did not have specific guidelines for the care of foreign patients (Table 1).

### Cultural competency, multicultural attitudes, multicultural efficacy, intercultural communicative competency, and hospital support for cultural competency

The average multicultural attitudes score was 72.69±8.82 (point average, 4.54±0.55), and the multicultural efficacy score was 67.51±8.46 (point average, 2.70±0.34), both reflecting above-moderate levels. The intercultural communicative competency score was 34.23±4.72 (point average, 3.42±0.47), and hospital support for cultural competency was 18.53±3.16 (mean rating, 2.32±0.40). The average score for overall cultural competency was 150.54±27.08 (point average, 4.56±0.82), indicating a moderate level (Table 2).

### Cultural competency by general characteristics

Significant differences in cultural competency were observed based on education level, religion, and overseas travel experience (Table 1). Participants holding a graduate degree exhibited significantly higher cultural competency compared to those with an associate or bachelor’s degree (F=5.47, *p*=.005). However, due to the limited number of participants with graduate degrees (n=10), these findings should be interpreted with caution. Additionally, participants with a religious affiliation showed significantly higher cultural competency than those without (t=2.32, *p*=.022). Similarly, individuals with overseas travel experience achieved significantly higher scores than those lacking such experience (t=2.62, *p*=.010).

### Correlations between cultural competency, multicultural attitudes, multicultural efficacy, intercultural communicative competency, and hospital support for cultural competency

Significant positive correlations were observed between cultural competency and several variables: multicultural attitudes (r=.65, *p*<.001), multicultural efficacy (r=.48, *p*<.001), intercultural communicative competency (r=.54, *p*<.001), and weakly with hospital support for cultural competency (r=.25, *p*=.002). These results indicate that higher levels of multicultural attitudes, multicultural efficacy, intercultural communicative competency, and hospital support for cultural competency are associated with increased cultural competency (Table 3).

### Factors influencing cultural competency

A hierarchical multiple regression analysis was conducted to identify factors that influence cultural competency. This analysis included significant general characteristics—education level, religion, and overseas travel experience—as well as independent variables that demonstrated significant correlations. These variables were multicultural attitudes, multicultural efficacy, intercultural communicative competency, and hospital support for cultural competency, all serving as predictors with cultural competency as the dependent variable. The Durbin-Watson statistic was 1.691, indicating an absence of autocorrelation. Tolerance values varied between .513 and .990, and variance inflation factors ranged from 1.010 to 1.951, suggesting that multicollinearity was not an issue.

Among general characteristics, variables that demonstrated significant differences in cultural competency, as well as those that showed significant correlations with it, were included in successive steps of the hierarchical analysis. In Step 1, general characteristics such as education level, religion, and overseas travel experience were entered. Step 2 involved the addition of individual factors, including multicultural attitudes, multicultural efficacy, and intercultural communicative competency, while controlling for the variables introduced in Step 1. Finally, Step 3 incorporated the organizational factor—hospital support for cultural competency—again controlling for the variables from Step 2. The results of the hierarchical multiple regression analysis, which aimed to identify factors influencing cultural competency among nurses in women’s hospitals, are presented in Table 4.

Model 1, the results of the analysis of variance for the regression model between general characteristics and cultural competency, accounted for 9.2% of the variance and was statistically significant (F=4.92, *p*<.001). Within the general characteristics, overseas travel experience was found to significantly influence cultural competency (β=.19, *p*=.016). However, education level (β=.15, *p*=.067) and religion (β=.15, *p*=.064) did not significantly impact cultural competency. This indicates that participants with overseas travel experience demonstrated higher cultural competency. Model 2 included individual factors such as multicultural attitudes, multicultural efficacy, and intercultural communicative competency. It revealed that multicultural attitudes (β=.47, *p*<.001) and intercultural communicative competency (β=.21, *p*=.013) positively affected cultural competency. Multicultural efficacy, however, did not show a significant effect (β=.10, *p*=.193). Model 2 accounted for 49.1% of the variance and was also statistically significant (F=23.03, *p*<.001). In Model 3, the addition of the organizational factor, specifically hospital support for cultural competency, indicated that hospital support did not significantly influence cultural competency (β=.08, *p*=.180). The factors that most significantly influenced cultural competency among nurses in women’s hospitals were multicultural attitudes (β=.46, *p*<.001) and intercultural communicative competency (β=.19, *p*=.025), in that order. The final model explained 49.8% of the variance and was statistically significant (F=20.11, *p*<.001).

## Discussion

This study investigated the levels of cultural competency among nurses in women’s hospitals, examining both individual and organizational factors that influence this competency. The results showed that nurses with more positive multicultural attitudes and higher intercultural communicative competency exhibited greater cultural competency.

In this study, the mean total score for participants’ cultural competency was 150.54 out of 231, with an average score of 4.56 out of 7. This was somewhat lower than the scores reported in previous studies [16, 20], which recorded average scores of 4.89 and 5.04, respectively, using the same instrument. This difference may stem from differences in workplace environments, as in the previous study, all [16] and 61% [20] of the participants were nurses from university hospitals and tertiary hospitals, which likely offer greater support for enhancing cultural competency. The subdomains were ranked from highest to lowest as follows: awareness, sensitivity, knowledge, and skills. This ranking aligns with the findings of Ko et al. [16], where awareness was scored highest, while sensitivity, knowledge, and skills were comparatively lower. Previous research has also shown that multicultural awareness is challenging to improve through short-term or one-time educational interventions [31]. The lower scores in knowledge and skills in this study could be due to the fewer participants who had received multicultural education. Most educational programs designed to enhance cultural competency among Korean nurses tend to concentrate on awareness and sensitivity, often neglecting knowledge and skills [32]. These findings underscore the necessity for comprehensive educational programs that encompass all dimensions of cultural competency.

Regarding the individual factors influencing cultural competency, both multicultural attitudes and intercultural communicative competency were identified as significant predictors. These factors retained their significance even in the final model, which incorporated the organizational factor. Multicultural attitudes emerged as the most influential individual factor (β=.46, *p*<.001), suggesting that higher levels of multicultural attitudes are associated with greater cultural competency. In this study, the average score for multicultural attitudes was 72.69 out of 96, with an average item score of 4.54 out of 6. This aligns with the score of 4.38 obtained using the same instrument in a previous study involving clinical nurses [21]. Although research exploring the correlation between multicultural attitudes and cultural competency among nurses is limited, studies involving nursing students have demonstrated that positive multicultural attitudes enhance cultural competency [10]. This supports the findings of the current study. Individuals with higher multicultural attitudes are more likely to view and embrace a multicultural society positively [33]. In contrast, negative attitudes towards multiculturalism can lead to discrimination, bias, and stereotypes, which pose barriers to nurses in delivering value-neutral care to individuals from diverse cultural backgrounds [34]. Therefore, it is essential for nurses to respect, accept, and empathize with cultural differences in their care delivery.

Another individual factor that influenced cultural competency was intercultural communicative competency (β=.19, *p*=.025); this finding is consistent with previous studies on healthcare professionals [22]. In this study, the average total score for intercultural communicative competency was 34.23 out of 50, with a mean rating of 3.42 out of 5. This is slightly lower than the score of 3.8 reported in earlier studies involving university students using the same instrument [35]. Additionally, 31.3% of the participants in this study had received multicultural education, compared to 58.5% in the university student study [35]. This suggests that exposure to multicultural education may enhance intercultural communicative competency. In nursing settings, where clear communication is crucial, improving intercultural communicative competency is vital for enhancing cultural competency [18]. Patient-centered communication when caring for multicultural patients can motivate patients to engage more actively in their treatment [36] and improve nurses’ clinical performance [17]. Conversely, nurses with lower communication competency may find it challenging and burdensome to connect with multicultural patients [14]. These nurses may also experience uncertainty and anxiety, which can negatively impact their cultural competency [37]. Given that nursing tasks—such as assessing patient needs, providing education, and explaining care—rely heavily on language, communication competency is crucial for patient care. However, there remains a shortage of training programs to enhance nurses’ intercultural communicative competency and a lack of resources or personnel support to address communication barriers in clinical settings [14]. Furthermore, with an increasingly diverse patient population in Korea, it is impractical for nurses to learn every patient’s native language. Therefore, medical institutions and government bodies should support the provision of interpreter services and multilingual guidelines. In addition to strengthening verbal communication skills, it is necessary to strengthen intercultural communicative competency through the development of nonverbal communication skills, such as understanding patients’ cultural backgrounds, communication attitudes based on consideration, and coping with cultural conflicts that arise during communication. To support this, communication-related nursing curricula should incorporate training aimed at enhancing intercultural communicative competency.

This study found that the mean total score for multicultural efficacy among individual factors was 67.51 out of 100, with a point average score of 2.70 out of 4. This is similar to the score of 2.65 reported in a previous study [20] that used the same tool to assess nurses’ multicultural efficacy. The subdomains were ranked as affective, practical, and cognitive, aligning with Byun and Park’s [20] findings, where the affective and practical domains scored higher than the cognitive domains. Although multicultural efficacy showed a positive correlation with cultural competency in this study, it did not emerge as a significant predictor in the final analysis. This finding contrasts with Byun and Park’s [20] study, which identified multicultural efficacy as a primary factor influencing nurses’ cultural competency. Beach et al. [38] suggested that multicultural attitude is an antecedent to multicultural efficacy, meaning that improvements in multicultural attitudes can enhance multicultural efficacy, which, in turn, positively impacts cultural competency. Since this study examined both multicultural attitudes and efficacy as individual factors, it limits the ability to assess the independent influence of each variable on cultural competency. When multiple variables with significant correlations are entered into a regression analysis, their relative impacts may obscure certain effects; in this case, the influence of multicultural attitudes on cultural competency was greater than that of multicultural efficacy, possibly rendering multicultural efficacy nonsignificant in the final model. Thus, future studies should examine each variable’s effect independently. Research on multicultural efficacy and cultural competency within Korean nursing remains limited, with only two studies, including this one. Moreover, because this study produced results that contrast with previous research [20], which identified multicultural efficacy as a major influencing factor on cultural competency, further investigation into the impact of multicultural efficacy on cultural competency—particularly among nurses in women’s hospitals frequented by multicultural patients—is necessary.

After examining the effect of organizational factor on cultural competency, the final model revealed that hospital support for cultural competency had no significant impact. The average total score for hospital support for cultural competency was 18.53 out of 32, with a mean rating of 2.32 out of 4. This is comparable to the 2.51 score reported in a previous study that used the same instrument to examine nurses at a general hospital [31]. However, over 80% of participants in this study reported that their hospital lacked guidelines for caring for foreign patients, which complicates comparisons with general hospitals where organizational support for multicultural patients is more readily available. Although there was a positive correlation between hospital support for cultural competency and cultural competency itself, it was not statistically significant in the final analysis. Conversely, a prior study found that programs and guidelines for foreign patient care within hospitals positively influenced nurses’ cultural competency [15]. Many MIW reside in smaller rural cities in Korea, where they primarily access local clinics or hospitals for maternity care [39]. Women’s hospitals, which provide care throughout women’s lives, especially need strong institutional support to improve the cultural competency of nurses serving multicultural patients. However, in regional small and mid-sized women’s hospitals, such as where this study was conducted, resources such as foreign language forms, training, and support for nurses working with multicultural patients were minimal or absent, likely contributing to the limited impact of hospital support on cultural competency in this context. The United States, with its long history as a multicultural society, has implemented extensive government-supported policies to reduce cultural conflicts between patients and healthcare providers. In contrast, Korea, with limited national support, primarily relies on a few large general hospitals with foreign patient centers and on-site interpreters to support multicultural patients [40]. Therefore, in addition to enhancing hospital support for cultural competency, government-led programs are essential to build cultural capacity, including training resources, particularly for nurses in small and medium-sized hospitals.

Finally, in the first model that examined the impact of general characteristics such as education level, religion, and overseas travel experience on cultural competency, it was determined that overseas travel experience plays a significant role (β=.19, *p*=.016). This indicates that individuals with more extensive overseas travel experience tend to have higher cultural competency, aligning with the findings of Yang et al. [6]. Overseas travel promotes multicultural acceptance [41], which in turn fosters greater openness and understanding of diverse cultures, thereby enhancing cultural competency [6]. However, as this study did not evaluate the frequency of overseas travel, further research is necessary to determine how different levels of travel experience may affect cultural competency.

This study utilized convenience sampling of nurses working in women’s hospitals in specific regions, which may restrict the applicability of the results to all nurses in women’s hospitals. Future studies should incorporate a broader and more varied group of nurses from different areas. Additionally, although this study included participants who had experience caring for foreign patients, it did not explore the frequency of these interactions, which limits the analysis of sensitivity to this factor. Furthermore, the study did not evaluate the participants’ personal multicultural backgrounds, such as whether they were from multicultural families or were MIW themselves. This omission restricts our understanding of cultural competency. Future research should consider these aspects to better assess cultural competency among nurses in women’s hospitals. The final model in this study accounted for 49.8% of the variance in cultural competency by including individual factors like multicultural attitudes and intercultural communicative competency. Therefore, further research should explore additional individual and organizational factors that could help explain the levels of cultural competency among nurses in women’s hospitals.

## Figures and Tables

**Table 1.** Differences in cultural competency according to participants’ general characteristics (N=150)

Characteristic	Categories	n (%)	Cultural competency	
			Mean±SD	t/F (*p*) Scheffé
Age (year)	Mean±SD, 37.15±8.42 (range, 23–58)			
	≥20, <30	35 (23.3)	143.11±26.28	2.22 (.089)
	≥30, <40	57 (38.0)	148.39±27.72	
	≥40, <50	41 (27.4)	157.68±28.15	
	≥50, <60	17 (11.3)	155.82±19.79	
Marital status	Single	65 (43.3)	146.57±28.45	1.58 (.117)
	Married	85 (56.7)	153.58±25.73	
Educational level	Associate degree^a^	78 (52.0)	148.90±24.49	5.47 (.005)^a,b<c^
	Bachelor’s degre^b^	62 (41.3)	148.32±27.64	
	≥Master’s degree^c^	10 (6.7)	177.10±31.40	
Religion	Yes	58 (38.7)	156.90±27.11	2.32 (.022)
	No	92 (61.3)	146.53±26.42	
Position in hospital	Staff nurse	120 (80.0)	147.92±27.47	2.93 (.057)
	Charge nurse	16 (10.7)	159.75±17.73	
	≥Head nurse	14 (9.3)	162.50±28.48	
Working unit	Delivery room	54 (36.0)	148.17±27.07	0.37 (.689)
	Neonatal unit	49 (32.7)	152.76±22.48	
	Obstetrics ward	47 (31.3)	150.96±31.48	
Overseas travel experience	Yes	129 (86.0)	152.83±27.17	2.62 (.010)
	No	21 (14.0)	136.48±22.24	
Having a foreign friend	Yes	16 (10.7)	159.38±38.27	1.01 (.328)
	No	134 (89.3)	149.49±25.41	
Ever received multicultural education	Yes	47 (31.3)	150.66±28.61	0.04 (.971)
	No	103 (68.7)	150.49±26.49	
Guidelines on caring for foreign patients in the hospital	Yes	27 (18.0)	151.11±21.38	0.12 (.904)
	No	123 (82.0)	150.41±28.25	

**Table 2.** Levels of cultural competency, multicultural attitudes, multicultural efficacy, intercultural communicative competency, and hospital support for cultural competency (N=150)

Variable	Mean±SD		Min	Max	Possible range
	Total score	Item score			
Cultural competency	150.54±27.08	4.56±0.82	67.00	213.00	33–231
Awareness	31.84±5.99	5.31±1.00	7.00	42.00	
Knowledge	31.03±7.00	4.43±1.00	10.00	48.00	
Sensitivity	54.87±12.19	4.57±1.02	24.00	81.00	
Skills	32.80±8.17	4.10±1.02	16.00	51.00	
Multicultural attitudes	72.69±8.82	4.54±0.55	51.00	95.00	16–96
Recognizing differences	29.41±3.01	4.90±0.50	22.00	36.00	
Openness and acceptance	21.28±3.46	4.26±0.69	13.00	30.00	
Commitment	22.01±3.62	4.40±0.72	14.00	30.00	
Multicultural efficacy	67.51±8.46	2.70±0.34	39.00	92.00	25–100
Cognitive	9.61±1.78	2.40±0.44	6.00	14.00	
Practical	30.69±5.30	2.56±0.44	13.00	45.00	
Affective	27.20±3.31	3.02±0.37	19.00	36.00	
Intercultural communicative competency	34.23±4.72	3.42±0.47	23.00	46.00	10–50
Hospital support for cultural competency	18.53±3.16	2.32±0.40	10.00	27.00	8–32

**Table 3.** Correlations between cultural competency and multicultural attitudes, intercultural communicative competency, multicultural efficacy, and hospital support for cultural competency (N=150)

Variable	Cultural competency	Multicultural attitudes	Multicultural efficacy	Intercultural communicative competency
Cultural competency	1			
Multicultural attitudes	.65 (<.001)	1		
Multicultural efficacy	.479 (<.001)	.496 (<.001)	1	
Intercultural communicative competency	.537 (<.001)	.565 (<.001)	.613 (<.001)	1
Hospital support for cultural competency	.249 (.002)	.248 (.002)	.097 (.236)	.253 (.002)

**Table 4.** Factors influencing cultural competency (N=150)

Variable	Cultural competency, β (*p*)		
	Model 1	Model 2	Model 3
General characteristics			
Educational level	.15 (.067)	.09 (.155)	.09 (.127)
Religion	.15 (.064)	–.06 (.383)	–.06 (.339)
Overseas travel experience	.19 (.016)	.09 (.139)	.09 (.146)
Individual factors			
Multicultural attitudes		.47 (<.001)	.46 (<.001)
Multicultural efficacy		.10 (.193)	.11 (.153)
Intercultural communicative competency		.21 (.013)	.19 (.025)
Organizational factor			
Hospital support for cultural competency			.08 (.180)
R^2^ (adjusted R^2^)	.092 (.073)	.491 (.470)	.498 (.473)
∆R^2^	.092	.400	.006
F (*p*)	4.92 (<.001)	23.03 (<.001)	20.11 (<.001)

The reference values were religion (0=No) and overseas travel experience (0=No).

## References

[1] Ministry of Justice (2022). 2022 Yearbook of Korea immigration statistics [Internet]. https://www.immigration.go.kr/immigration/1570/subview.do;jsessionid=DSWxdDK2kmp-e6aBjA_rVBOzbnIkywXGhXyUiFiR.wizard-19-rlv5j?enc=Zm5jdDF8QEB8JTJGYmJzJTJGaW1taWdyYXRpb24lMkYyMjglMkY1NzI1MDclMkZhcnRjbFZpZXcuZG8lM0Y%3D.

[2] Lim HS (2011). The experience of transition in pregnancy and childbirth among the married immigrant women in Korea. Korean J Women Health Nurs.

[3] Park H, Park M, Chun Y (2015). A study on education needs related to prenatal care programs in married immigrant women. J Korea Acad-Ind Coop Soc.

[4] Kim MJ (2011). A comparative study on birth outcomes between Korean women and immigrant women. Korean J Women Health Nurs.

[5] Park KS, Shin HJ (2011). A study on the factors influencing on cultural competence of university students. J Reg Stud.

[6] Yang SO, Kwon MS, Lee SH (2012). The factors affecting cultural competency of visiting nurses and community health practitioners. J Korean Acad Community Health Nurs.

[7] Kim SH, Kim KW, Bae KE (2014). Experiences of nurses who provide childbirth care for women with multi-cultural background. J Korean Public Health Nurs.

[8] Cross TL (1989). Towards a culturally competent system of care: a monograph on effective services for minority children who are severely emotionally disturbed.

[9] Kang HJ, Lim EM (2012). Validation study of multicultural attitude scale for Korean university students. Asian J Educ.

[10] Kim N, Lim SY (2014). Relationships between multicultural awareness, multicultural attitude and multicultural efficacy among nursing students. Multicult Educ Stud.

[11] Yang HJ, Byun EK (2019). Factors affecting multicultural efficacy of nurse. Asia-Pac J Multimed Serv Converg Art Humanit Sociol.

[12] Koh CK, Koh SK (2009). Married female migrants’ experiences of health care services. J Korean Acad Soc Nurs Educ.

[13] Park HS, Ha SJ, Park JH, Yu JH, Lee SH (2014). Employment experiences of nurses caring for foreign patients. J Korean Acad Nurs Adm.

[14] Jang HY, Lee E (2016). Caring experiences of the nurses caring for foreign inpatients of non-english speaking. J Korea Acad-Ind Coop Soc.

[15] Chae DH, Park YH, Kang KH, Lee TH (2012). A study on factors affecting cultural competency of general hospital nurses. J Korean Acad Nurs Adm.

[16] Ko MS, Choi KO, Choi EH (2016). A study on the relationship of cultural competence, self efficacy and job stress in nurses caring for hospitalized foreign patients. J Korean Clin Nurs Res.

[17] Ryu YJ, Lee YM (2021). Influence of cultural competency and intercultural communication on clinical competence of emergency unit nurses caring for foreign patients. J Korean Crit Care Nurs.

[18] Jang MM, Jang SH, Lee DY (2022). Factors affecting the cultural competence of general hospital nurses: self-efficacy and intercultural communication skill. J Korean Soc Multicult Health.

[19] Lee J, Lee H, Kim S, Jang Y (2014). Comparison of perceived nurses’ cultural competence of nurses and foreign patients. J Korean Clin Nurs Res.

[20] Byun HS, Park MK (2020). Impacts of multicultural acceptability, transcultural self-efficacy on the cultural competency among nurses. J Health Info Stat.

[21] Kim M (2019). A study on multicultural attitude, cultural competence and influencing factors among nurses in Korea. J Humanit Soc Sci.

[22] Kwon SA, Yang NY, Song MS, Kim NY (2016). Healthcare workers’ cultural competence and multi-cultural job stress. J Korean Acad Soc Home Care Nurs.

[23] Chae DH, Lee CY (2014). Development and psychometric evaluation of the Korean version of the cultural competence scale for clinical nurses. Asian Nurs Res.

[24] Munroe A, Pearson C (2006). The Munroe multicultural attitude scale questionnaire: a new instrument for multicultural studies. Educ Psychol Meas.

[25] Oh WO, Park ES, Suk MH, Im YJ (2016). Development and psychometric evaluation of the transcultural self-efficacy scale for nurses. J Korean Acad Nurs.

[26] Jeffreys MR (2000). Development and psychometric evaluation of the transcultural self-efficacy tool: a synthesis of findings. J Transcult Nurs.

[27] Lee DW (2011). A comparative study of intercultural communicative competence of Korean students as sojourners and American students as hosts in American universities. Korean J Commun Stud.

[28] Lee EM, Kim SH (2015). Variables affecting the intercultural communication skills of nursing students. Korean Parent Child Health J.

[29] Balcazar FE, Suarez-Balcazar Y, Taylor-Ritzler T (2009). Cultural competence: development of a conceptual framework. Disabil Rehabil.

[30] Choi YK, Ahn JW, Kim KS (2018). Influence of cultural competency, intercultural communication, and organizational support on nurses’ clinical competency caring for foreign patients. Health Soc Welf Rev.

[31] Choi KS, Morgan S, Thongpriwan V, Lee SY, Jun M (2014). A proposed teaching model to improve cultural competency care for undergraduate Korean nursing students. J Korean Acad Soc Nurs Educ.

[32] Son HM, Je MJ, Yi BJ (2014). Integrative review on cultural competence of nurse. Korean J Cult Arts Educ Stud.

[33] Jang I, Lee K (2014). Mediating effect of multicultural attitude between multicultural perception and multicultural efficacy of elementary school teachers. Multicult Peace.

[34] Cho JY, Youn MS (2015). A study on attitude toward immigrants and multicultural efficiency among nursing college students. J Korean Soc Multicult Health.

[35] Yoo H, Kim K, Lee H, Park B, Park S, Park J (2019). Cultural capacity and intercultural communication of university students in nursing. J Korean Acad Rural Health Nurs.

[36] Yhi B (2016). The lived experiences of clinical nurses who working at the women’s hospital caring for foreign wives in Korea: a narrative study approach. J Korea Acad Ind Coop Soc.

[37] Ahn JW, Jang HY (2019). Factors affecting cultural competence of nurses caring for foreign patients. Health Policy Manag.

[38] Beach MC, Price EG, Gary TL, Robinson KA, Gozu A, Palacio A (2005). Cultural competence: a systematic review of health care provider educational interventions. Med Care.

[39] Statistics Korea (2022). A marriage to a foreigner by city/county [Internet]. https://kosis.kr/statHtml/statHtml.do?orgId=101&tblId=DT_1BB0002&conn_path=I2.

[40] https://doi.org/10.23170/snu.000000025723.11032.0000435.

[41] Song M (2010). The factors influencing multicultural receptivity in Korea. Minjok Yeonku.

